# Draft Genome Sequence of *Tumebacillus* sp. Strain BK434, Isolated from the Roots of Eastern Cottonwood

**DOI:** 10.1128/MRA.00351-20

**Published:** 2020-05-28

**Authors:** Dana L. Carper, Christopher W. Schadt, Leah H. Burdick, Udaya C. Kalluri, Dale A. Pelletier

**Affiliations:** a Biosciences Division, Oak Ridge National Laboratory, Oak Ridge, Tennessee, USA; Portland State University

## Abstract

A Gram-positive bacterium was isolated from the root of an eastern cottonwood tree (Populus deltoides) in Georgia and identified as a *Tumebacillus* species with 99% 16S rRNA nucleotide identity to *Tumebacillus avium*. The genome is 4.6 Mbp and encodes 4,072 proteins and 251 RNAs.

## ANNOUNCEMENT

*Tumebacillus* is a genus of Gram-positive rod-forming bacteria that was only recently described in the phylum *Firmicutes* (1). Since its discovery, eight species from this genus have been isolated from diverse environments, such as permafrost (1), soil (2, 3), river water (4), algal scum (5), and the gut of a vulture (6). Here, we report the first draft genome of a *Tumebacillus* species associated with a plant root.

The roots from a Populus deltoides (genotype WV94) (7) tree growing on a nursery site in Bellville, Georgia, were sampled in 2018. Fine roots (<2 mm) were excised from freshly harvested root samples and processed as described previously (8, 9). Nonsterilized roots were macerated in 10 ml of MgSO_4_ (10 mM) and serially diluted onto Reasoner’s 2A (R2A) agar (10). Cultures were isolated through three rounds of restreaking onto R2A medium at 28°C. The isolate was inoculated into R2A liquid medium and grown for 2 days at 30°C. Genomic DNA was isolated with the Qiagen DNeasy blood and tissue kit according to the manufacturer’s instructions. An initial identification was carried out using Sanger sequencing of the 16S rRNA amplicon of strain BK434 with primers 27F and 1492R (11). Based on an NCBI BLAST (12) search of the nonredundant/nucleotide database, the 16S rRNA amplicon of strain BK434 was found to have 99.15% nucleotide identity, across 1,414 bp, to *Tumebacillus avium.* Because the genus *Tumebacillus* has very few genome-sequenced representatives, we proceeded with genome sequencing to allow greater exploration of the genetic potential of this group. The draft genome of *Tumebacillus* was generated at the U.S. Department of Energy (DOE) Joint Genome Institute (JGI) using Illumina technology (13). A standard shotgun library was constructed; briefly, DNA was sheared by ultrasonication (Covaris, Woburn, MA) to a 600-bp average fragment size, and an Illumina shotgun library was prepared with the Kapa Biosystems library preparation kit (Roche, Wilmington, MA) and sequenced using the NovaSeq Xp reagent kit v1.0 with 2 × 151-bp reads on the Illumina NovaSeq platform, which generated 9,668,990 reads totaling 1,460,017,490 bp. Default parameters were used for all software unless otherwise specified. Raw Illumina reads were quality filtered to remove known sequencing artifacts and contaminants and read depth was normalized using BBTools (14). The filtered reads were assembled using SPAdes (v3.12.0) (phred-offset, 33; cov-cutoff, auto; t, 16; m, 64; careful; k, 25,55,95) (15), and for quality purposes, contigs of <1 kbp were discarded (BBTools reformat.sh: minlength). The final draft assembly contained 34 contigs (*L*_50_, 3; *N*_50_, 708,514 bp) in 33 scaffolds, totaling 4,646,936 bp with a GC content of 57.94%. The final assembly was based on 1,431,753,368 bp of Illumina data with a mapped coverage of 305.7×. The genome was annotated using the IMG Microbial Genome Annotation Pipeline (MGAP) (v4.16.4) (16). A total of 4,072 protein-coding genes were predicted, with 251 RNA genes and 7 CRISPR arrays. Protein-coding regions included those involved in spore germination, which is common in *Firmicutes*, and in flagellar motion. Flagella have been characterized for some species of *Tumebacillus* (5, 17), including *Tumebacillus avium* (6), the strain that is 99.15% similar based on 16S rRNA findings.

### Data availability.

The assembly has been deposited in GenBank under accession number SLXS00000000 and is also available from the IMG/M database under accession number 2795386103. Raw sequences have been deposited in the NCBI Sequence Read Archive under BioProject number PRJNA520069 and run number SRR8861549.
